# Percutaneous embolization for a subacute gastric fistula following laparoscopic sleeve gastrectomy: a case report and literature review

**DOI:** 10.1186/s12893-020-00896-4

**Published:** 2020-10-08

**Authors:** Hung-Hsuan Yen, Yu-Ting Lin, Jin-Ming Wu, Kao-Lang Liu, Ming-Tsan Lin

**Affiliations:** 1 Department of Surgery, National Taiwan University Hospital Hsin-Chu Biomedical Park Branch, Hsinchu County, Taiwan; 2 Department of Surgery, National Taiwan University Hospital, Taipei, Taiwan; 3 Department of Medical Imaging, National Taiwan University Cancer Center, National Taiwan University Hospital, National Taiwan University College of Medicine, Taipei, Taiwan

**Keywords:** Fistula, Leak, Sleeve gastrectomy, Bariatric surgery, Embolization

## Abstract

**Background:**

The management for subacute or chronic fistula after bariatric surgery is very complicated and with no standard protocol yet. It is also an Achilles’ heel of all bariatric surgery. The aim of this case report is to describe our experience in managing this complication by percutaneous embolization, a less commonly used method.

**Case presentation:**

A 23-year-old woman with a body mass index of 35.7 kg/m^2^ presented with delayed gastric leak 7 days after laparoscopic sleeve gastrectomy (LSG) for weight reduction. Persistent leak was still noted under the status of nil per os, nasogastric decompression, and parenteral nutrition for 1 month; therefore, endoscopic glue injection was performed. The fistula tract did not seal off, and the size of pseudocavity enlarged after gas inflation during endoscopic intervention. Subsequently, we successfully managed this subacute gastric fistula via percutaneous fistula tract embolization (PFTE) with removal of the external drain 2 months after LSG.

**Conclusions:**

PFTE can serve as one of the non-invasive methods to treat subacute gastric fistula after LSG. The usage of fluoroscopy-visible glue for embolization can seal the fistula tract precisely and avoid the negative impact from gas inflation during endoscopic intervention.

## Background

The risk of leak after laparoscopic sleeve gastrectomy (LSG) was 2.4, and 89% of leak occurred at the proximal third of the stomach [1]. Most leaks result from the disruption of blood supply around the angle of His combined with increased intraluminal pressure and decreased gastric tube compliance after LSG [2]. Re-laparoscopy or even re-laparotomy is necessary for leak happening within 24 to 48 h after primary bariatric surgery or anytime when uncontrolled septic signs develop and persist; otherwise, a non-operative management is suggested by most bariatric surgeons [3]. The management for subacute or chronic leak after bariatric surgery with fistula tract formation is more complicated, with no standard protocol yet, and also an Achilles’ heel of all bariatric surgery [4–9].

## Case presentation

The 23-year-old woman with the body mass index (BMI) of 35.7 kg/m^2^ was evaluated for LSG. Her past medical history was significant for bilateral knee osteoarthritis and had taken over-the-counter pain relievers for a long time with suboptimal effects. The blood tests from her primary care physician denoted prediabetes, borderline hypercholesterolemia, and mild elevations of the liver function tests (glycated hemoglobin 6.4%, total cholesterol 220 mg/dL, aspartate aminotransferase 39 U/L, and alanine aminotransferase 64 U/L). Preoperative workups, including contrast-enhanced computed tomography (CT) scan and abdominal sonography, further reported a moderate fatty liver. She had also attempted to lose weight through different methods, including hypocaloric diet and exercise, for many years but without success. Therefore, the patient was indicated for and underwent LSG [10].

The LSG was performed using a 36-Fr bougie as a stent, and the staple line was oversewn to prevent postoperative leak and hemorrhage. A 7-mm silicone closed wound vacuum (CWV) drain system was placed intraoperatively along the staple line with its tip at the left esophagogastric junction (EGJ). The course of hospitalization was uneventful and the patient was discharged on postoperative day (POD) 4.

**Fig. 1:**
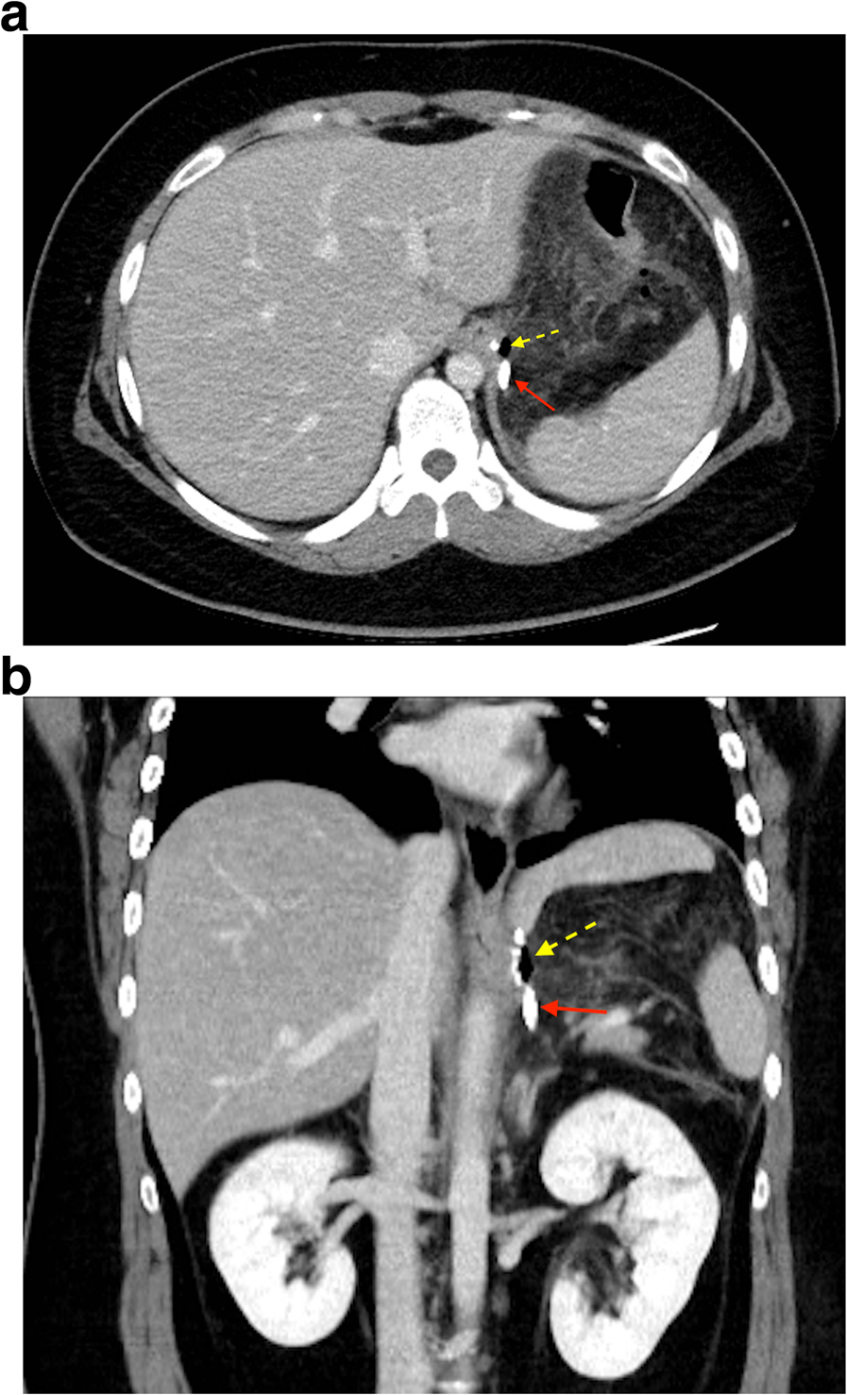
CT scan, axial (**a**) and coronal (**b**) views, at POD7. The minor leak with a small air-pocket was denoted by the dashed arrow, and there was some fat-stranding nearby but no obvious fluid accumulation. The solid arrow indicated the tip of the CWV drain placed intraoperatively. Abbreviation: POD, postoperative day; CT, computed tomography; CWV, closed wound vacuum

**Fig. 2:**
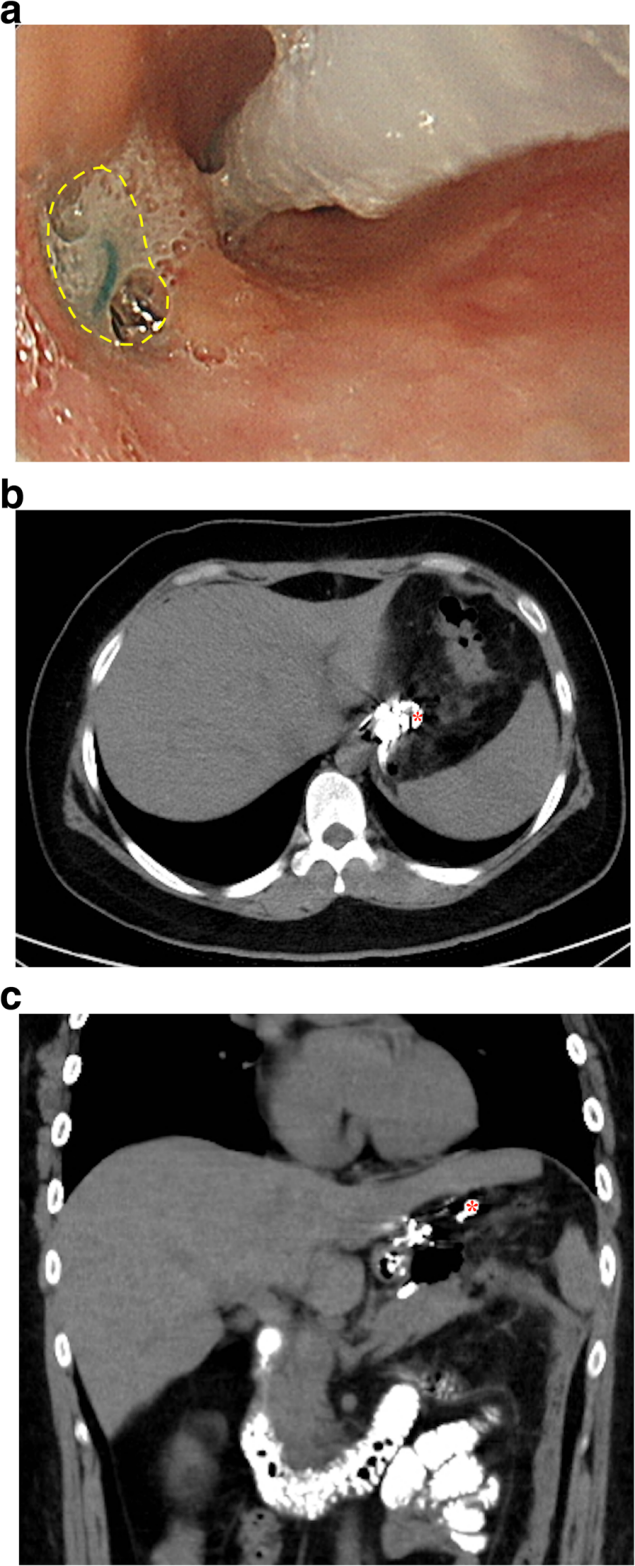
Endoscopic glue injection for closure of the fistula at POD40 (**a**), and an oral contrast enhanced CT scan one week, at POD48, after endoscopic glue injection (**b** and **c**). An about 5 mm fistula tract opening was found via endoscopic examination (dashed line, **a**), and was closed by Histoacryl® and Lipiodol® injection. The following oral contrast enhanced CT scan one week later still showed a minor leak (asterisk, **b** and **c**) and a more enlarged size of the pseudocavity. Abbreviation: POD, postoperative day; CT, computed tomography

However, the patient presented with mild fever, upper abdominal pain, and purulent discharge from the CWV drain on POD7. The CT scan showed a minor leak at left EGJ (Fig. 1). After risks and benefits of surgical exploration for leak repair in this situation were provided to the patient, non-operative management with broad-spectrum antibiotics, nasogastric tube decompression, and external drainage via the previously inserted CWV drain was adopted [3]. Meanwhile, considering the prolonged duration of nil per os and to provide an adequate nutritional support, total parenteral nutrition (TPN) was given based on the American Society for Parenteral and Enteral Nutrition clinical guidelines for hospitalized adult patients with obesity [11]. The nutritional weight calculation was performed as follows: “nutritional weight = ideal body weight (BW) + 0.25 ∗ (actual BW – ideal BW)” [12]. The estimated energy expenditure was 25 kcal/kg/day, and the calorie composition of TPN solution was customized with 48% glucose, 18% amino acid, and 34% fat emulsion. Supplementation of multivitamins and mixtures of trace elements (including zinc, copper, manganese, chromium, and iodine) was given daily if not otherwise contraindicated. Complete blood count, serum electrolytes, liver and renal functions were monitored weekly. On POD40, endoscopic examination with n-butyl-2-cyanoacrylate (Histoacryl®, B. Braun) and ethiodized oil (Lipiodol®, Guerbet) injection in a 1:1 mixture (Fig. 2a) was performed because of the persistent minor leak with daily drainage amount < 3 ml [13, 14]. But, unfortunately, an oral contrast enhanced CT scan on POD48 still showed an unresolved leak with a more enlarged pseudocavity (Fig. 2b and c).

**Fig. 3:**
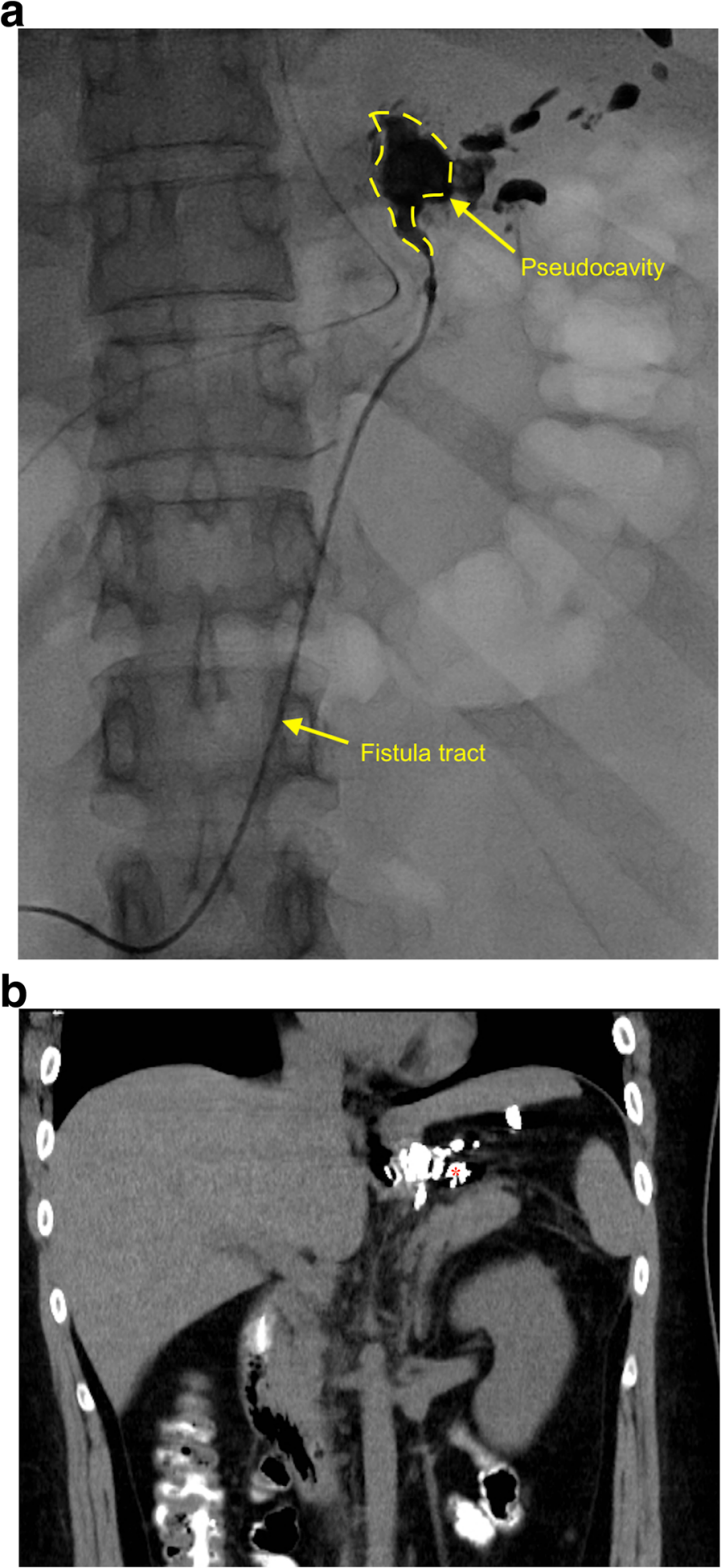
Percutaneous fistula tract embolization was performed through the CWV drain insertion site at POD63 (**a**) and an oral contrast enhanced CT 5 days later, at POD68, showed the space of pseudocavity was filled with glue and no obvious oral contrast leak was noted (**b**). Abbreviation: CWV, closed wound vacuum; POD, postoperative day; CT, computed tomography

In this situation, other alternative managements, including surgical exploration, esophageal stent, over-the-scope clip (OTSC), through-the-scope clip (TTSC), and percutaneous fistula tract embolization (PFTE), were explained in detail and provided to the patient. After careful consideration and discussion, the patient selected and received PFTE on POD63. A guidewire was inserted through the CWV drain, and then a 40-cm 5-Fr KMP catheter (COOK® Medical) was introduced after the removal of CWV drain. After the pseudocavity and fistula tract were well illustrated under fluoroscopy, the glue (33%, Histoacryl®: Lipiodol® = 1: 2) was slowly and continuously injected followed by gradual withdrawal of the catheter (Fig. 3a) [13, 14]. The follow-up oral contrast enhanced CT scan on POD68 showed the pseudocavity was sealed with glue and there was no more leak (Fig. 3b). The patient resumed oral intake since POD75 and was discharged on POD86. She had a BMI of 27 and no clinical signs of gastric leak or recurrence of the fistula in the most recent follow-up on POD183.

## Discussion and conclusions

In the present report, we provided a successful experience in treating a subacute gastric fistula 2 months after LSG via PFTE with removal of the external drain following an initial failure of endoscopic glue injection 1 month after LSG. We used a fluoroscopy-visible glue to guide the embolization and avoided using esophagogastroduodenoscopy simultaneously to reduce the negative impact from gas inflation.

We chose PFTE to treat subacute gastric fistula for its simplicity, effectiveness, non-invasiveness, and also low cost, based on the technique and successful experiences shared by Assalia et al. [9] However, we noticed a total of 50 sessions of biological glue embolization were performed for their 23 patients with a median of 2.2 sessions per patient and 61% of all their patients required more than one application [9]. In our opinion, there are two points that would possibly improve the success rate and reduce the number of treatment session. First of all, the timing is crucial. In the early stage of gastric leak, the inflammation and infection are ongoing and the formation of pseudocavity and fistula tract has not been mature enough; therefore, at this timing, the application of glue is not able to securely seal either the fistula opening, cavity, or tract [15]. Secondly, the fibrin glue used by Assalia et al. was not illustratable on fluoroscopy, so a combined endoscopy is necessary to ensure the adequate injection of the glue. Nevertheless, gas inflation during endoscopy may worsen the pseudocavity and fistula tract as shown in our patient (Figs. 1 and 2). We added Lipiodol® to the glue (Histoacryl®), and in this manner, we were able to visualize the glue injection during the whole process of embolization by fluoroscopy to ensure the precise and adequate sealing [13, 14]. Thus, we could also avoid the potentially negative effect of gas inflation during endoscopic intervention. The inclusion of 10 (42%) patients with “acute” gastric fistula and the combination of endoscopic visualization (gas inflation) may explain the more treatment sessions per patient required in the study of Assalia et al. [9]

On the other hand, we did not choose endoscopic stenting or clipping, such as esophageal stent [4], OTSC [5], and TTSC [6], for two reasons. Firstly, even though esophageal stent, OTSC, and TTSC could yield a successful rate of 73 and 80% for leak after LSG respectively, there was 1% of major bleeding, 28% of stent migration, and rare complications such as esophageal rupture, tracheoesophageal fistula, or aortoesophageal fistula that could cause a catastrophic consequence [4, 5, 16]. Secondly, dealing with adverse events after elective procedure, such as bariatric surgery, is always more complicated, especially when there has been no standard method of management. The decision differs greatly between healthcare systems, costs of the instruments, patient’s preferences, and patient-physician’s communications. Endoscopically primary closure of the gastric fistula had also been considered for our patient with a 5-mm dehiscence; however, it was not performed concerning the high requirement of technique [8]. Recently, endoscopic internal drainage by a double-pigtail stent with prompt mobilization of the external drainage has become a promising management for hemodynamically stable patients with early leak after LSG with a successful rate of 92.8% [7]. However, whether this management is also effective for subacute or chronic gastric fistula has not been validated yet.

PFTE with fluoroscopy-visible glue materials may serve as an alternative non-invasive management for subacute gastric fistula after LSG. Nonetheless, whether this method is with high success rate requires further validation from a larger case number.

## Data Availability

Not applicable.

## Abbreviations

LSG: Laparoscopic sleeve gastrectomy
PFTE: Percutaneous fistula tract embolization
BMI: Body mass index
CT: Computed tomography
CWV: Closed wound vacuum
EGJ: Esophagogastric junction
POD: Postoperative day
TPN: Total parenteral nutrition
BW: Body weight
OTSC: Over-the-scope clip
TTSC: Through-the-scope clip
